# Expression of Clementine Asp-Rich Proteins (CcASP-RICH) in Tobacco Plants Interferes with the Mechanism of Pollen Tube Growth

**DOI:** 10.3390/ijms23147880

**Published:** 2022-07-17

**Authors:** Luigi Parrotta, Lavinia Mareri, Iris Aloisi, Claudia Faleri, Gaetano Distefano, Alessandra Gentile, Angela Roberta Lo Piero, Verena Kriechbaumer, Marco Caruso, Giampiero Cai, Stefano Del Duca

**Affiliations:** 1 Interdepartmental Center for Agri-Food Industrial Research, University of Bologna, 40126 Bologna, Italy; luigi.parrotta@unibo.it (L.P.); iris.aloisi2@unibo.it (I.A.); stefano.delduca@unibo.it (S.D.D.); 2 Department of Biological, Geological and Environmental Sciences, University of Bologna, 40126 Bologna, Italy; 3 Department of Life Sciences, University of Siena, 53100 Siena, Italy; lavinia.mareri@unisi.it (L.M.); claudia.faleri2@unisi.it (C.F.); 4 Department of Agricultural, Food and Environment, University of Catania, 95131 Catania, Italy; distefag@unict.it (G.D.); gentilea@unict.it (A.G.); rlopiero@unict.it (A.R.L.P.); 5 Plant Cell Biology, Oxford Brookes University, Oxford OX3 0BP, UK; vkriechbaumer@brookes.ac.uk; 6 Council for Agricultural Research and Economics, Research Centre for Olive, Fruit and Citrus Crops, Acireale, 95024 Catania, Italy; marco.caruso@crea.gov.it

**Keywords:** actin filaments, ASP-RICH protein, calcium, cell wall, ROS

## Abstract

Low-molecular-weight, aspartic-acid-rich proteins (ASP-RICH) have been assumed to be involved in the self-incompatibility process of clementine. The role of ASP-RICH is not known, but hypothetically they could sequester calcium ions (Ca^2+^) and affect Ca^2+^-dependent mechanisms. In this article, we analyzed the effects induced by clementine ASP-RICH proteins (CcASP-RICH) when expressed in the tobacco heterologous system, focusing on the male gametophyte. The aim was to gain insight into the mechanism of action of ASP-RICH in a well-known cellular system, i.e., the pollen tube. Pollen tubes of tobacco transgenic lines expressing CcASP-RICH were analyzed for Ca^2+^ distribution, ROS, proton gradient, as well as cytoskeleton and cell wall. CcASP-RICH modulated Ca^2+^ content and consequently affected cytoskeleton organization and the deposition of cell wall components. In turn, this affected the growth pattern of pollen tubes. Although the expression of CcASP-RICH did not exert a remarkable effect on the growth rate of pollen tubes, effects at the level of growth pattern suggest that the expression of ASP-RICH may exert a regulatory action on the mechanism of plant cell growth.

## 1. Introduction

Pomelo (*Citrus maxima* [Burm.] Merrill) and other citrus accessions are characterized by a gametophytic self-incompatibility (SI) system based on S-RNase [1,2]. Analysis of putative homologs of key genes and proteins of known SI systems has already allowed several genes involved in pollen tube reject to be identified, including RNase, F-box, S-phase kinase-associated protein1 (SKP1)-like, S1 SI locus-linked pollen 3.15, ubiquitin-activating enzyme E1, DELLA, and Ca^2+^-binding protein genes [3,4]. In addition to the above-mentioned genes, three uncharacterized genes (i.e., cit.11563, cit.5456, and cit.5776 in the Affymetrix Citrus Gene Chip) are strongly upregulated concomitantly with pollen tube growth arrest in the self-incompatible clementine variety “Comune” [3,5]. These genes cluster in a region of approximately 11 kb and encode for three proteins containing 79, 53, and 74 amino acids, respectively [3], enriched in aspartic acid residues, thus named *Citrus clementine* (Hort. ex Tan) aspartic acid-rich proteins (CcASP-RICHs). Although homologs of the CcASP-RICH have been identified in other species, their function has not been characterized yet; however, preliminary studies suggest an involvement in Ca^2+^ homeostasis [3].

Since CcASP-RICHs are new proteins that could be involved in the mechanism of self-incompatibility, it is important to investigate how they work. To investigate the mechanism of action at the cellular level, *CcASP-RICH* genes were expressed in the tobacco heterologous system and the effect analyzed in the pollen tube. The pollen tube is a tip-growing cell in which the apical region is enriched with Golgi-derived secretory vesicles that fuse with the plasma membrane, providing new lipids, proteins, and cell wall materials to the growing tube. The apical domain contains ions, small molecules, and proteins that polarize the pollen tube [6,7]. The apical polarization affects the arrangement of the cytoskeleton, which in turn regulates the trafficking of organelles and vesicles in the pollen tube [8]. Most of these processes are strictly controlled and regulated by complex networks of signaling events [9,10,11], with calcium (Ca^2+^) playing a leading role. Ca^2+^ regulates, integrates, and coordinates pollen tube growth, and an oscillatory tip-focused Ca^2+^ gradient is essential for pollen germination and pollen tube elongation [12]. In addition to being a signaling molecule, Ca^2+^ is also required for structural organization of the cell wall by cross-linking pectins, thus increasing cell wall stiffness behind the apex [12,13]. In addition to the Ca^2+^ gradient, other ion gradients exist in a growing pollen tube, such as K^+^, Cl^−^, and H^+^ [14], as well as an apical pool of reactive oxygen species (ROS) [15].

To understand how CcASP-RICH could affect cell growth, a transgenic line of tobacco (*Nicotiana tabacum* L.) expressing *CcASP-RICH* was analyzed. Because the mechanism of action of ASP-RICHs is unclear, we performed an extensive analysis by visualizing the distribution of calcium ions, ROS, and protons, as well as actin filament organization, cell wall deposition, and the pattern of pollen tube growth. The results indicate that the expression of ASP-RICHs does not alter the pollen tube growth rate, but it does affect the growth pattern, thereby resulting in loss of pulsed growth. In addition, alterations in Ca^2+^ distribution and the actin filament arrangement were found with consequences for the deposition of cell wall components.

## 2. Results

### 2.1. Analysis of CcASP-RICH Presence and Expression in Transgenic Tobacco Pollen Tubes

**Figure 1:**
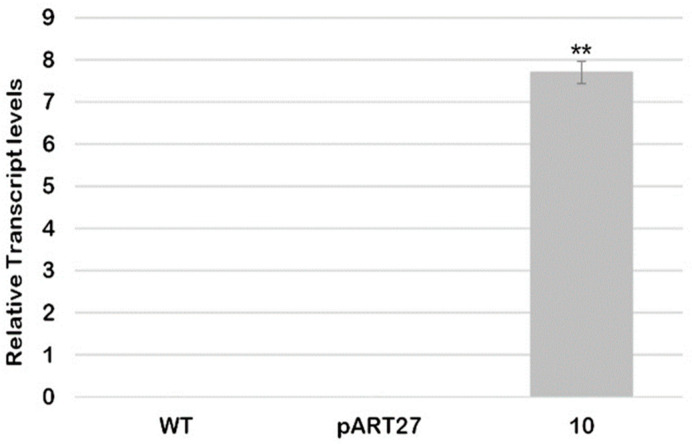
Relative transcript level of CcASP-RICH in the pollen of WT and transgenic lines. Expression values were normalized to the EF1α reference gene. Bars with SD are the mean of the three replicates. Asterisks indicate statistically significant differences (** = for *p* < 0.01).

The presence of the *CcASP-RICH* gene and its relative expression in pollen from line 10 were analyzed by qRT-PCR. As expected, no signal was detected in both WT and pART27 lines, whereas consistent expression was reported in line 10 (Figure 1).

### 2.2. Pollen Viability Is Affected by the Expression of CcASP-RICH but Not Pollen Tube Elongation

**Figure 2:**
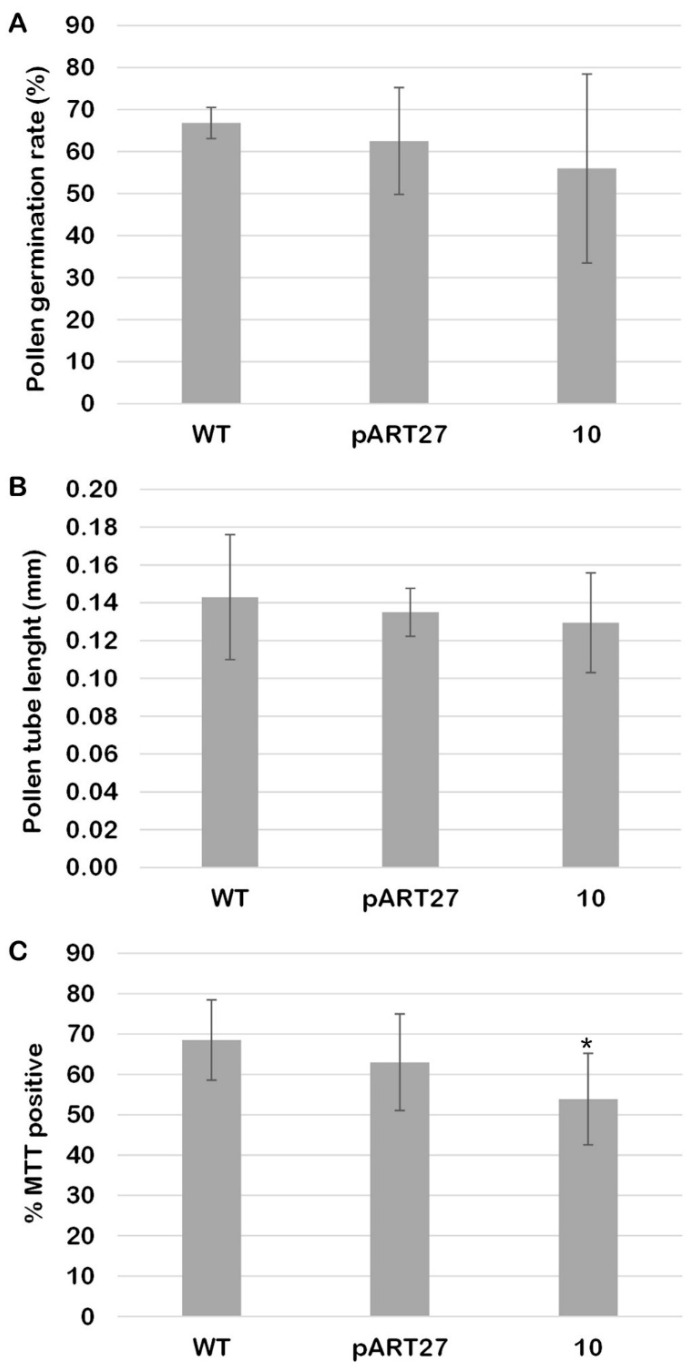
Viability of pollen grains, germination rate, and tube length of at least 100 pollen grains for each line analyzed. (**A**) Germination rate, expressed as percentage, of pollen grains. (**B**) Length of pollen tubes germinating from pollen grains after 3 h of germination. (**C**) Percentage of MTT positive pollen grains. Sample sets were compared with one-way ANOVA. Asterisks indicate statistically significant differences (* *p* < 0.05).

CcASP-RICHs did not significantly affect the ability of pollen grains to germinate; compared to the WT line, the pollen grains of line 10 showed no significant reduction in germination rate (Figure 2A). In addition, the pollen tube length after 180 min of germination was not statistically different (Figure 2B). In addition, pollen viability was assessed using MTT (2,5-diphenyl tetrazolium bromide) staining, highlighting significant differences between WT pollen grains and pollen grains from line 10 (Figure 2C).

### 2.3. Calcium Ion Profile Is Altered in Pollen Tubes Expressing CcASP-RICH

**Figure 3:**
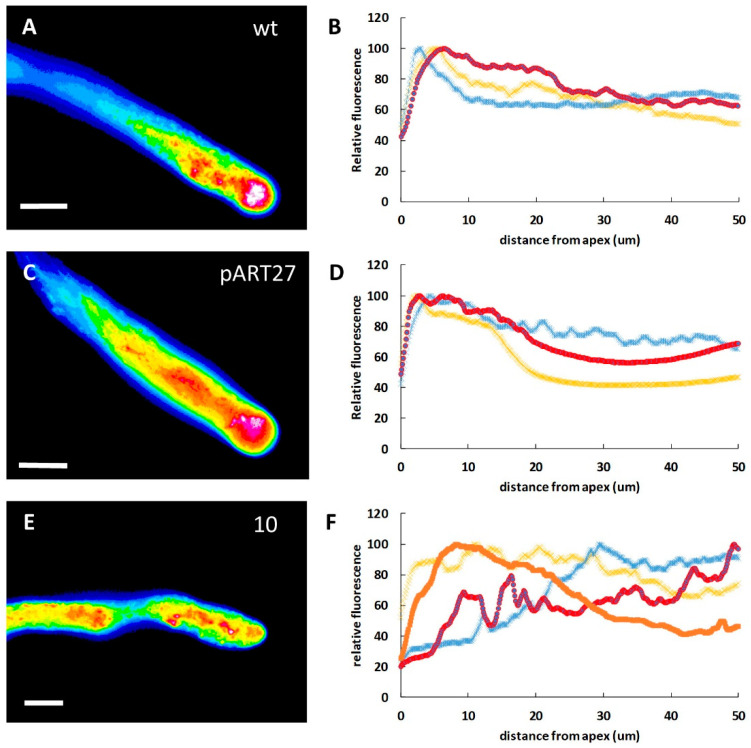
Distribution of cytosolic Ca^2+^ in WT and transgenic lines of tobacco pollen tubes. (**A**) False-color visualization of cytosolic Ca^2+^ in WT pollen. (**B**) Three measurements (in red, blue, and yellow) of the cytosolic Ca^2+^ gradient in WT pollen. In all graphs, the *y*-axis shows the relative fluorescence intensity, while the *x*-axis is the distance from the tube apex expressed in µm. (**C**) Ca^2+^ distribution in pART27 line. (**D**) Measurements of relative fluorescence intensity in pART27 line. Three examples of measurements (in red, yellow, and blue) are shown. (**E**) Cytosolic Ca^2+^ distribution in transgenic line 10. (**F**) Relative fluorescence measurements in line 10. In this case, four measurements (in red, yellow, blue, and orange) are shown. Bars: 10 µm.

The cytoplasmic calcium level was assessed using the Fluo-4/AM fluorescent probe. The probe turned out to be a very simple and consistent tool for studying the level of calcium. As is widely discussed in the literature, the protocol is technically easy and reliable. In WT lines, cytosolic Ca^2+^ consistently accumulated at the apex of pollen tubes (Figure 3A) and returned to basal values 10 μm from the apex (graph in Figure 3B). The tobacco pART27 line showed a Ca^2+^ distribution comparable to the WT line (Figure 3C,D). In contrast, Ca^2+^ distribution was significantly altered in transgenic line 10. In this case, Ca^2+^ was distributed differently among the individual pollen tubes (Figure 3E). Some pollen tubes showed a partial Ca^2+^ gradient, whereas others exhibited Ca^2+^ accumulations in the distal regions of pollen tubes (Figure 3F). Thus, some variability was present among the analyzed pollen tubes.

### 2.4. ROS Levels Do Not Differ between WT and Transgenic Lines

**Figure 4:**
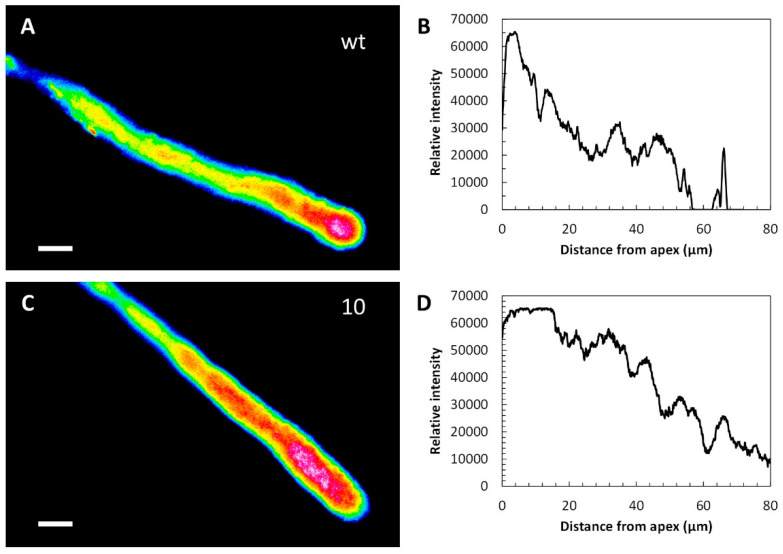
Visualization of ROS in pollen tubes of WT and transgenic line 10. (**A**) ROS levels in a representative pollen tube. (**B**) ROS distribution is confirmed by measurements. (**C**) ROS distribution in a representative transgenic pollen tube, line 10. (**D**) Measurements in line 10 confirmed no significant differences from WT. Bars: 10 µm.

In pollen tubes from the WT line, ROS levels were much more pronounced in the apical region (Figure 4A,B). In this case, transgenic line 10 (Figure 4C,D) showed no substantial difference in ROS levels compared with the WT line, although ROS levels in line 10 appeared to decrease more slowly than in the WT sample. Therefore, the expression of CcASP-RICHs did not apparently affect ROS distribution. The graphs show measurements for individual pollen tubes, but the ROS profile was very similar in all pollen tubes investigated.

### 2.5. The pH Gradient in the Pollen Tube Is Partially Compromised

**Figure 5:**
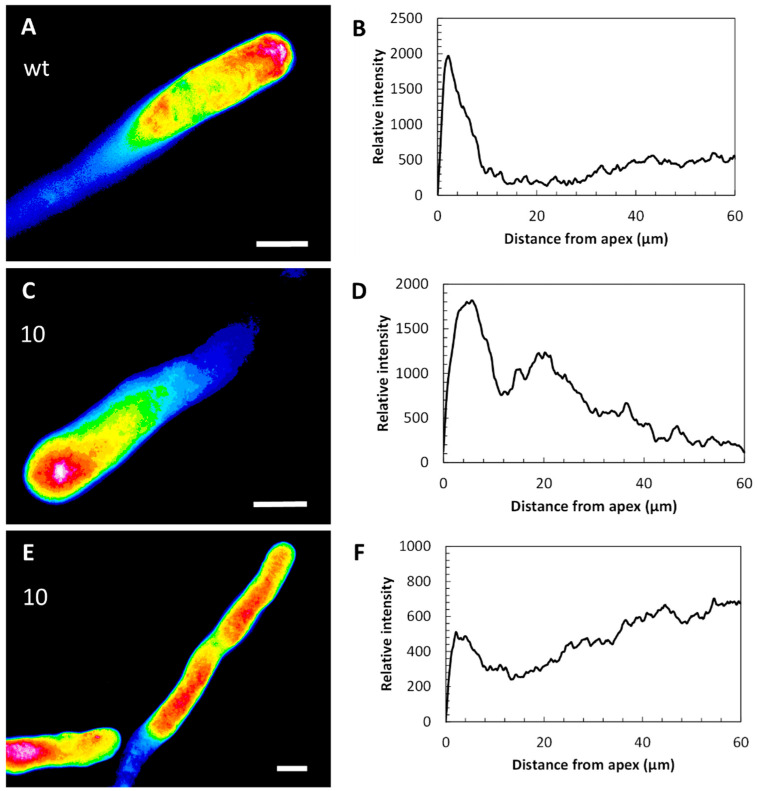
Effects of CcASP-RICH on the proton gradient in pollen tubes. (**A**) In WT pollen, protons accumulate at the tube apex. (**B**) Measurement of relative fluorescence intensity confirmed the apical distribution. (**C**) Example of proton distribution in line 10: the pH is still acidic at the apex, as revealed by measurements (**D**). (**E**) In other cases, proton distribution was deeply altered and more homogeneous, as evidenced by the fluorescence measurement (**F**). Bars: 10 µm.

WT pollen tubes were characterized by low pH values at the apex (Figure 5A, red), which was also confirmed by relative fluorescence intensity measurements (Figure 5B). In transgenic line 10, the proton gradient was more heterogeneous. Some pollen tubes showed a normal proton distribution (Figure 5C,D), although the decrease from the apex was less pronounced. Instead, other pollen tubes (nearly 50%) were characterized by a more homogeneous proton redistribution in the pollen tube (Figure 5E,F), with low pH regions scattered throughout the pollen tube. The graphs show measurements of individual pollen tubes, but the profile was virtually equivalent in the observed cases.

### 2.6. Actin Distribution Differs between WT and Transgenic Pollen Tubes

**Figure 6:**
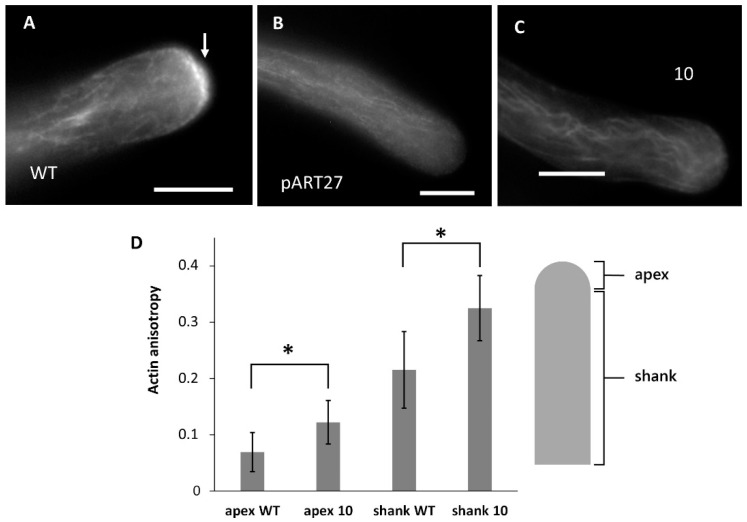
Distribution of actin filaments in WT pollen tubes and in pollen tubes expressing CcASP-RICH. (**A**) WT pollen tube with the typical longitudinal distribution of actin filaments and actin fringe (arrows). (**B**) Pollen tube expressing pART27 with a longitudinal array of actin filaments. (**C**) Actin filaments in line 10. Here, curved wavy bundles are observable, indicating substantial damage to actin filaments. (**D**) Analysis of actin filament anisotropy in pollen tubes from WT and transgenic line 10. Analyses were carried out in two distinct regions, the hemispherical apex, and the tube shank. Comparing actin anisotropy in the apical region between WT and line 10 shows statistically significant differences, as well as in the tube shank. The *y*-axis shows the relative anisotropy scale. Asterisks indicate statistically significant samples (*p* < 0.05). Bars: 10 µm.

In WT pollen tubes, actin filaments were distributed longitudinally according to the growth axis (Figure 6A). A slight accumulation of actin could often be observed in the apical or immediately subapical region (arrow), corresponding to the actin fringe. The pART27 sample showed regular, longitudinally oriented actin filaments (Figure 6B). The organization of the actin cytoskeleton was severely impaired in transgenic line 10 (Figure 6C). Actin filaments were frequently intermingled and clustered together. In general, actin filaments were not linearly arranged.

Anisotropy analysis of actin distribution was performed in the apical hemispherical dome, and separately in the pollen tube stem, over a length of approximately 100 μm (Figure 6D). Anisotropy in the apical hemispherical region increased slightly, but significantly, between the WT line and transgenic line 10. This significant difference also resulted when comparing actin filaments in the pollen tube stem. By assuming that the anisotropy involves altered actin filament dynamics, we hypothesized that the expression of CcASP-RICH in transgenic line 10 results in increased longitudinal stabilization of actin filaments, both at the apex and in the shank.

### 2.7. CcASP-RICH Affects the Distribution of Newly Secreted Cell Wall Material but Not Callose

**Figure 7:**
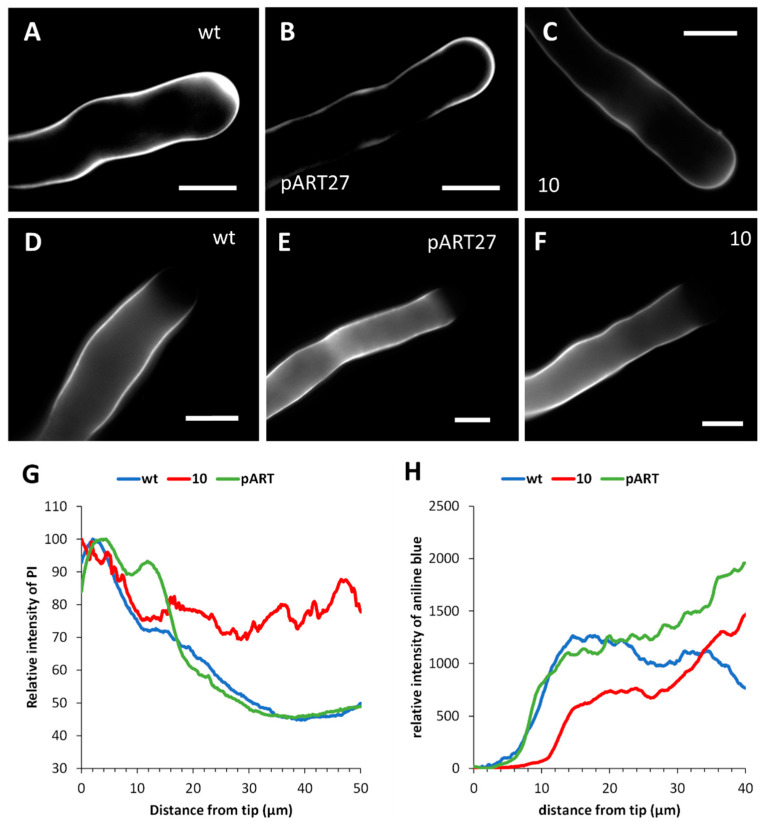
Distribution of newly secreted cell wall material. (**A**) WT pollen tube with accumulation of cell wall material at the apex. (**B**) Pollen tube expressing pART27 shows a fluorescence signal at the apex. (**C**) Pollen tube of line 10 showing more homogeneous distribution of new cell wall material. Bars: 10 µm. (**D**) WT pollen tube with accumulation of callose in the shank and absence in the apex. (**E**) Pollen tube expressing pART27 shows a pattern of callose similar to the control. (**F**) Pollen tube of line 10; callose is absent in the apex and is evenly distributed in the shank. Bars: 10 µm. (**G**) Graphs of the distribution of new cell wall material. WT pollen tubes are comparable to pART27 but not to pollen tubes from transgenic line 10. (**H**) Graphs of the relative amount of callose at the edge of pollen tubes. In all cases, a very weak signal is present in the first 5–10 μm from the apex; then the amount of callose increases progressively, although with different trends in distinct samples.

Given the changes in actin organization in transgenic line 10, the distribution of newly secreted cell wall material was then studied. We made use of the fluorescent PI probe, which is not highly specific for pectins. The latter are usually labeled using antibodies against different pectin classes; however, here we preferred to use a fluorescent probe to be applied to live cells in order to obtain a dynamic view of the secretion of new cell wall material. In the WT line, fluorescence accumulation in the apical region was observable, indicating that this is the area where the new cell wall material is actively secreted (Figure 7A). A similar distribution pattern was also observed in the pART line (Figure 7B). In contrast, some substantial differences were observed in transgenic line 10 (Figure 7C), where new secreted material did not consistently accumulate in the apical region. To obtain a quantitative visualization, fluorescence was measured along the edge of pollen tubes from the tube tip toward the grain for approximately 50 μm (Figure 7G). The WT line sample (blue) highlighted the typical accumulation profile of new secreted material in the first 5 μm from the apex. The pART line (green) showed a profile overlapping with the control sample. In contrast, transgenic line 10 (red) showed a more homogeneous profile along the edge of pollen tubes, while it differed from the control and pART samples in the first 20 μm from the tip.

Unlike pectins, callose was uniformly present along the pollen tube in the WT line, except in the apical region (Figure 7D). This distribution pattern was comparable to the pART line (Figure 7E) and transgenic line 10 (Figure 7F). Quantitative analysis of relative fluorescence along the edge of pollen tubes also revealed no differences (Figure 7H).

### 2.8. The Growth Pattern of Pollen Tubes Is Altered by the Expression of CcASP-RICH

**Figure 8:**
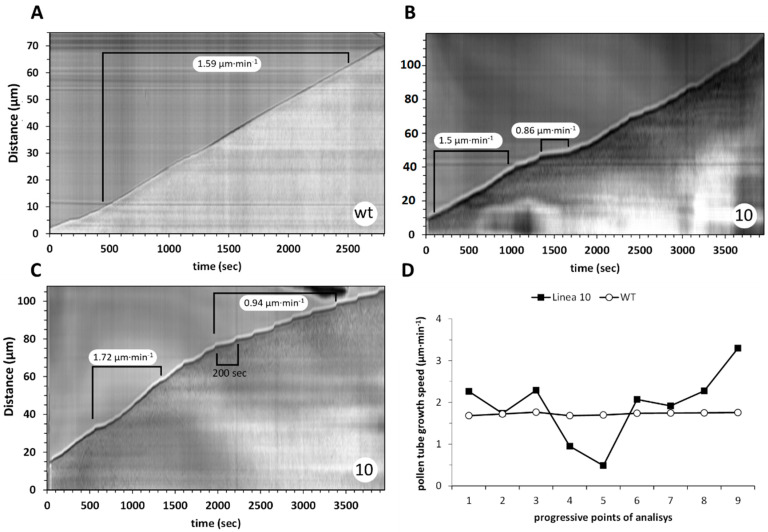
Kymograph analysis of pollen tubes from WT and transgenic line 10. (**A**) Kymograph of WT pollen tube, which grows constantly and does not show significant changes in growth rate over the analyzed time. The *y*-axis shows the distance expressed in micrometers, while the *x*-axis reports the analysis time in seconds. (**B**) Pollen tube of line 10. The unevenness of the growth profile is clear and characterized by periods of regular velocity interspersed with low-speed periods (at least 50% reduction). (**C**) Another kymograph example of pollen tubes of line 10. Again, an irregular trend is observed with an initial part characterized by the standard growth rate followed by a second phase with a significantly decreased growth speed. In this case, the typical “step” (200 s) between two fast growth peaks can be observed. (**D**) Pollen tube growth rate analysis; data refer to a single WT pollen tube and a single line 10 pollen tube but are representative of the results obtained. The data refer to a series of nine progressive points, each spaced 10 min apart. While velocity is constant in WT, pollen tubes of line 10 behave differently from each other, but all show a growth rate characterized by increases in velocity mixed with sharp decreases.

Both germination and cytological data suggested that the mechanism underlying pollen tube growth may be altered by CcASP-RICH expression. We investigated this possibility by kymograph analysis, which allows point velocity data to be obtained. WT pollen tubes showed a linear and constant growth profile (Figure 8A) with velocities of approximately 1.6 µm·min^−1^. Pollen tubes of line 10 showed a heterogeneous and altered growth pattern. Figure 8B,C show two examples in which the behaviors of pollen tubes from transgenic line 10 were not identical. Figure 8B shows a pollen tube with strong decreases in growth rate, down to 0.86 µm·min^−1^, and then increasing again. In the case of Figure 8C, after an initial steady growth, the pollen tubes experienced a decrease in growth rate with velocities down to 1 µm·min^−1^ (Figure 8C). However, the overall growth rate of pollen tubes was not affected. WT pollen tubes maintained a constant growth rate (approximately 1.5 µm·min^−1^), whereas pollen tubes of transgenic line 10 showed strong fluctuations in growth rate (Figure 8D).

### 2.9. CcASP-RICH Does Not Affect Callose Plug Deposition or Even Generative Cell Movement

**Figure 9:**
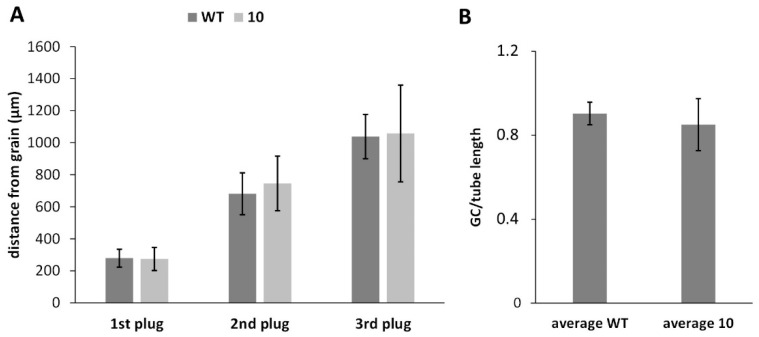
(**A**) Analysis of callose plug deposition in WT pollen tubes and transgenic line 10. Data are reported as the ratio of distance of callose plugs from the grain to pollen tube length. No significant differences were found for the three callose plugs analyzed. At least 20 pollen tubes of identical length were counted. Sample sets were compared statistically by one-way ANOVA, and no differences were shown. (**B**) Generative cell movement (GC) in relation to pollen tube length. Data were calculated as the ratio of distance traveled by generative cells to pollen tube length. No significant difference, analyzed by one-way ANOVA, was found between WT and line 10 samples.

Because pollen tube growth is characterized by periodic deposition of callose plugs, which help to maintain a constant turgor pressure and save the energy required to keep the cytoplasm alive, we monitored the deposition frequency of the first three callose plugs. No significant differences were found between pollen tubes of WT and line 10 (Figure 9A). The only difference was that the second and mostly the third callose plug of line 10 were deposited with less precision than the WT. The second parameter analyzed was the distance covered by the generative cell in relation to the pollen tube. Again, we showed no significant difference between WT and line 10 samples during 7 h of monitoring (Figure 9B).

## 3. Discussion

Despite the identification of specific S-RNase (S_11_) in clementine, a complete view of the key genes involved in citrus SI is still unclear. Different candidate genes putatively involved in pollen–pistil interactions and SI have been identified in several citrus species such as pomelo, mandarin, and clementine [2,3,4,5,16]. Among a set of candidate genes involved in pollen–pistil interactions and SI, three uncharacterized genes, encoding for CcASP-RICH proteins, were strongly expressed during the SI response in Clementine [3]. In our previous work [17], *CcASP-RICH* were overexpressed in *Nicotiana tabacum* “Wisconsin 38” and revealed differences with WT in terms of pollen grain quantity and viability. Specifically, mutant lines expressing *CcASP-RICH* exhibited defects in anther morphology and poor pollen production. In addition, pollen showed defects in germination. CcASP-RICH fused to a fluorophore at the C-terminus (GFP, Supplementary Figure S1A,B) or N-terminus (mCherry, Supplementary Figure S1C,D), respectively, showed a transient expression in tobacco leaf epidermal cells. Both CcASP-RICH-GFP and CcASP-RICH-mCherry showed cytosolic localization (Supplementary Figure S1B). Overexpression of *CcASP-RICH* in the tobacco system resulted in no visible change to the endoplasmic reticulum or the actin cytoskeleton (Supplementary Figure S1C,D).

The role of CcASP-RICH cannot be deduced from sequence homology analysis because BLAST alignments identify several ESTs with unknown functions belonging to the genus *Citrus*. The encoded proteins are acidic because aspartic amino acids predominate (i.e., ASP-RICH). A putative role of acidic proteins has been suggested in clams where they are key components of the calcification process, possibly being involved in nucleation, inhibition, and orientation of crystal growth [18]. This is because Asp-/Glu or poly-Asp/poly-Glu-rich domains can bind high amounts of Ca^2+^ [18]. The finding that CcASP-RICH can bind calcium ions is partly supported by biochemical evidence previously obtained using the Stains-All dye [17], which showed that CcASP-RICH can exhibit calmodulin-like behavior and thus bind calcium ions.

To gain a better understanding of the role of CcASP-RICHs, their genes were expressed in tobacco plants, and their effect was evaluated in the pollen tube, considered as a model cell. An interesting fact was obtained by transient expression of CcASP-RICHs in epidermal cells of tobacco leaves; the data indicated that the cytosol is the cellular compartment where CcASP-RICHs accumulate. Because ASP-RICHs presumably can bind Ca^2+^, our first analysis was addressed to determine the effects on the distribution of Ca^2+^ in the pollen tube. We hypothesized that CcASP-RICHs may interfere with the mechanism that controls pollen tube growth, as it is based on the uneven distribution of Ca^2+^ in the pollen tubes [19]. Ca^2+^ enters the apical region of the pollen tube through the activity of either stretch, voltage-gated, CNGCs, or GLRs Ca^2+^ channels [20,21] and is likely expelled into the cell wall or stored in pollen tube organelles [22]. In addition, Ca^2+^ can also bind to cell wall polysaccharides, such as pectins, or even to plasma membrane-associated glycoproteins, such as arabinogalactan proteins (AGPs) [23]. While pectins use Ca^2+^ to create cross-links between adjacent molecules, thereby strengthening the cell wall, AGPs can function as a so-called Ca^2+^ capacitor (storage) [24]. In both cases, pectins and AGPs can take part in regulating pollen tube growth by controlling Ca^2+^ availability. The data in this work indicates that the expression of CcASP-RICH alters the distribution of Ca^2+^ in pollen tubes. The transgenic line exhibited profound alterations in the Ca^2+^ gradient, causing Ca^2+^ to be more homogeneously distributed. Given that the oscillatory accumulation of Ca^2+^ at the pollen tube apex correlates with the oscillation between fast and slow growth phases [25,26], it follows that any alteration in Ca^2+^ levels significantly impact the way the pollen tube grows.

In plants, reactive oxygen species (ROS) are a consequence of aerobic metabolism but are also involved in functions such as pathogen defense and cell signaling. In pollen tubes, ROS are correlated to the Ca^2+^ gradient by acting as positive feedback for Ca^2+^ accumulation [27,28,29,30]. In root hairs of *Arabidopsis thaliana*, ROS have been proposed to modulate tip growth by activating Ca^2+^ channels [15]. Accumulation of ROS in the tube apex may result from either intense metabolism or from ROS production by plasma membrane enzymes [15,31]. ROS function at the tube apex is still controversial [32]; most likely they regulate cell growth by interfacing with Ca^2+^ flux. In transgenic pollen tubes expressing CcASP-RICH, we found no significant alterations in ROS levels. On one hand, this emphasizes that the action of CcASP-RICH might be specific for Ca^2+^; on the other hand, this indicates that CcASP-RICH-induced changes in Ca^2+^ levels do not have a notable effect on ROS production, suggesting a direct effect on binding Ca^2+^ rather than an indirect effect on its influx or efflux.

In contrast to ROS levels, the intracellular pH profile in pollen tubes appeared to be affected by CcASP-RICH expression leading to a more homogeneous distribution of protons. The role of the pH gradient in pollen tubes is not fully elucidated [33,34,35]; apparently, the proton gradient is a consequence of growth rather than an anticipation. Protons at the tube apex could result from the removal of methyl groups in cell wall pectins with the production of acidic pectins; the latter, before complexing with Ca^2+^, lose protons that could be transported within the pollen tube [36]. Proton influx to the apex could be used to regulate actin filament dynamics [34,37]. Indeed, some actin filament regulatory proteins are controlled by pH [35,38]. The evidence that CcASP-RICH influences the distribution of Ca^2+^, and thus protons, suggests a correlation between the two processes. According to accepted models, the Ca^2+^ peak at the tube apex anticipates growth, while the proton peak follows growth [39].

Although Ca^2+^ and proton flux occur at two distinct times during pollen tube growth, both processes regulate actin filament dynamics. This explains the differences in actin filament distribution between WT and transgenic lines. The higher anisotropy in the transgenic line, both in the apex and shank, suggests that changes in Ca^2+^ and proton distribution make actin filaments less dynamic. Since successful growth of pollen tubes is linked to proper actin filament dynamics [40,41], CcASP-RICHs could therefore affect actin-related processes, e.g., transport of secretory vesicles.

Effects at the level of vesicular transport could result in less secretion of new cell wall material. Indeed, the data indicate a lesser accumulation of newly secreted material, presumably methyl-esterified pectins, in the pollen tube growth zone. This will certainly affect the mechanical strength of the cell wall by making it stiffer and in turn affect pollen tube growth [36]. The impact on secretion does not appear to affect callose production; we hypothesize that the enzyme callose synthase [42,43] accumulates at the apex in a sufficient amount to maintain a constant and adequate level of callose. No effects were observed on the deposition of callose, even at the level of the callose plugs and therefore on the movement of the generative cell (which is synchronized with the deposition of plugs), suggesting once again that the deposition of callose is not compromised.

## 4. Conclusions

Although the expression of CcASP-RICH in tobacco pollen does not have a dramatic effect on pollen tube growth, alteration of Ca^2+^ distribution and intracellular pH, actin filaments, and secretion actually affect the growth rate. The most consistent result is the appearance of very different growth rates that fluctuate dramatically in a matter of minutes; therefore, evidence suggests that the expression of CcASP-RICHs has important effects on the growth mechanism of the pollen tube. The latter is not constant in the transgenic line (as opposed to WT), indicating damage at the level of the growth regulation system. We can assume that the expression of CcASP-RICHs affects the level of cytosolic calcium and, in turn, the organization of actin filaments. Changes in the organization of actin affect the deposition of new cell wall material, thereby altering the stiffness of the cell wall and hindering the growth of pollen tubes. The data obtained, albeit preliminary, indicate that the presence of ASP-RICHs alters the normal functioning of a plant cell, influencing some of the basic processes such as calcium levels and cell wall deposition. Therefore, ASP-RICHs could be considered as a controller of cellular functions and thus represent a new, as yet uncharacterized, potential regulatory agent.

## 5. Materials and Methods

### 5.1. Plant Material and Transgenic Line Construction

The *CcASP-RICH*1 gene, represented by the probe set cit.11563 in the Affymetrix Citrus GeneChip described in Caruso et al. [3], was overexpressed into *Nicotiana tabacum* “Wisconsin 38”. The ORFs were cloned into a pART27 vector containing the CaMV 35S promoter. *Agrobacterium*-mediated transformation was performed following the protocol described by Benekos and collaborators [44] using the GV3101 strain. PCR was used to confirm transformation with primers annealing to CaMV 35S and to the *CcASP-RICH* ORFs. One transgenic line, named 10, the wild type (WT), and the empty pART27 line were used for the experiments reported below. 

### 5.2. Analysis of CcASP-RICH Presence and Expression in Transgenic Tobacco Pollen Tubes

Mature pollen grains were collected from line 10 and the pART27 line, and total DNA was extracted from 15 mg pollen grains using the CTAB method with minimal modifications [45]. DNA concentration and purity were determined spectrophotometrically using a Qubit spectrophotometer (Thermo Scientific, Wilmington, DE, USA) [46]. DNA integrity was evaluated by electrophoresis separation on 0.8% agarose gel stained with ethidium bromide (1 μg mL^−1^). The presence of the transgenic gene was assayed by qRT-PCR. qRT-PCR was performed in the Bio-Rad iQ5 (Bio-Rad, Hercules, CA, USA) in 20 μL reactions containing 10 μL of SsoAdvancedTM Universal SYBER^®^ Green Supermix with ROX (Bio-Rad, Hercules, CA, USA), 0.4 μL of each primer [3] (10 μM), 5 μL of DNA (2 ng·μL^−1^), and 4.2 μL of water. PCR conditions were: 95 °C/3 min followed by 95 °C/15 s, 60 °C/30 s, and 72 °C/30 s for 40 cycles. Three biological and two technical replicates for each sample, along with two negative controls per plate, were performed. 

For gene expression analysis, total RNA was extracted from 15 mg of pollen grains using the Trizol™ method (Invitrogen, Carlsbad, CA, USA) following the manufacturer’s instructions. Genomic DNA was removed by DNase treatment (AMPD1-DNAse I Amplification Grade, Sigma-Aldrich, St. Louis, MI, USA). RNA was quantified using a Qubit spectrophotometer (Thermo Fisher Scientific, Wilmington, DE, USA), and its quality was assessed by non-denaturing 1.2% agarose gel. The complementary DNA (cDNA) was synthesized from 500 ng of total RNA using the iScriptTM cDNA Synthesis Kit (Bio-Rad, Hercules, CA, USA) according to the manufacturer’s protocol. Expression of *Cc-Asp-Rich* genes was determined using qRT-PCR on a Bio-Rad iQ5 (Bio-Rad, Hercules, CA, USA). The qRT-PCR reaction (20 μL) consisted of gene-specific primers Cit.11563.1.S1_at [3], SsoAdvancedTM Universal SYBER^®^ Green Supermix (Bio-Rad, Hercules, CA, USA), and the template. Thermal cycling conditions were 95 °C/30 s followed by 95 °C/15 s, 60 °C/30 s, and 72 °C/30 s for 40 cycles. Three biological and two technical replicates for each sample, along with two negative controls per plate, were performed. The relative expression levels were calculated by the ΔCt method using Elongation Factor 1 α (EF1α) as a reference for normalization [47].

### 5.3. Pollen Growth Measurement

Tobacco plants were grown under standard conditions in greenhouses at a constant temperature of 25 °C with a 16 h/8 h light/darkness photoperiod with a PPFD (photosynthetic photon-flux density) of 350 μmol·m^−2^·s^−1^, with relative humidity of 60% ± 10%, and ambient CO_2_ concentration. Pollen was collected from opening flowers. Pollen was dehydrated and stored at −20 °C. When needed, pollen was hydrated at room temperature overnight in a moist chamber, and then the pollen was placed in Petri dishes containing BK medium supplemented with 12% sucrose [48]; pollen was incubated at room temperature under constant and slow stirring. We measured the germination rate, length, and viability of at least 100 pollen tubes after 3 h of germination to statistically evaluate all parameters as well as the length of germinated tubes using ImageJ software v. 1.53k (Wayne Rasband and contributors, National Institute of Health, Bethesd, MD, USA, https://imagej.nih.gov/ij/index.html, accessed on 13 June 2022) to evaluate the growth rate. Pollen viability was checked by MTT (2,5-diphenyl tetrazolium bromide). The test solution contained a 1% concentration of the MTT substrate in 5% sucrose. After 15 min incubation at 30 °C, the pollen samples were visualized under a light microscope.

### 5.4. Detection of Ca^2+^, pH, and ROS in Pollen Tubes

For cytoplasmic Ca^2+^ analysis, germinated pollen was incubated for 15 min at 4 °C with 0.5 μM Fluo-4/AM probe dissolved in a solution containing 2% cetyltrimethyl ammonium bromide (CTAB), 100 mM Tris-HCl, and 40 mM EDTA, with a pH value of 8.0 [41]. Samples were observed within 1–2 min. Proton distribution was monitored with the BCECF probe, AM (2′,7′-Bis-(2-carboxyethyl)-5-(6′)-carboxyfluorescein, acetoxymethylester) [49]. Images were taken within 3–5 min after probe addition. ROS were visualized with the fluorescence probe 2′,7′-dichloro-dihydrofluorescein diacetate (H2DCFDA), at a final concentration of 10 μM [50]. Samples were observed within 1–2 min.

In all cases, images were taken with a Zeiss AxioImager fluorescence microscope equipped with an Axiocam MRm camera and 63× objective. For more intuitive visualization of Ca^2+^, pH, and ROS levels, images were converted to a 16-color scale using ImageJ software (Image menu > Lookup Table > 16 colors). For all three analyses, the fluorescence signal was measured from the apex of pollen tubes for at least 50 µm down to the pollen grain after scale calibration.

### 5.5. Confocal Microscopy

Leaf epidermal samples were imaged using a Zeiss PlanApo 100×/1.46 NA oil immersion objective on a Zeiss LSM880 confocal microscope equipped with an Airyscan detector (Carl Zeiss AG, Jena, Germany). Typically, 512× images were collected in 8-bit with 4-line averaging with excitation at 488 nm (GFP) and 561 nm (RFP/mCherry), and emissions at 495–550 nm and 570–615 nm, respectively.

### 5.6. Visualization of Actin in the Pollen Tube

Actin filaments were visualized according to the protocol of Lovy-Wheeler et al. [51]. Actin filament orientation in pollen tubes was assessed by the ImageJ FibrilTool plugin, which allows quantitative description of fiber anisotropy and its average orientation in cells [52]. For pollen tubes, two distinct areas were selected: the hemispherical dome (approximately 10 µm from the apex), and the shank of the pollen tube. Analysis was performed on at least 10 different pollen tubes, with equivalent lengths randomly selected for all case studies.

### 5.7. Labeling of Cell Wall Components and of Callose Plugs

Labeling of material secreted into the cell wall was performed by staining with propidium iodide (PI) [53]. Callose was visualized using aniline blue [54]; to visualize callose plugs, staining was performed after pollen tubes were allowed to grow for 7 h. Fluorescence measurement of PI and aniline blue was performed along the edge of the pollen tube (from the apex toward the granule) using the ImageJ “segmented line” tool with a selection width of approximately 2 µm. ImageJ was also used to measure the distance of the first three callose plugs from the pollen grain.

### 5.8. Kymograph Analysis

Videos of pollen tubes were analyzed to determine the growth profile. Videos were approximately 1 h long after pollen had germinated for 1 h in BK medium + 12% sucrose. For analysis, pollen was germinated in 24-well plates coated with polylysine to prevent pollen movement in the medium during video recording. A Nikon phase-contrast inverted light microscope was used with a 40× objective. Video recordings were captured with Pinnacle Media Center software v. 4 (http://www.pctvsystems.com/, Hauppauge Digital Europe, L-2540 Luxembourg, accessed on 2 May 2022) in MPEG-2 format. Subsequently, videos were converted to uncompressed AVI format using the free Virtual Dub software v. 1.10 (http://virtualdub.org/, accessed on 12 April 2022). The resulting files were loaded into ImageJ and scanned with the kymograph plug-in created by J. Rietdorf (FMI Basel) and A. Seitz (EMBL Heidelberg) (https://www.embl.de/eamnet/html/body_kymograph.html, accessed on 5 May 2022). For each frame, gray values were measured along a region of interest (ROI) manually specified by the operator. Starting from the gray values, a new image (kymograph) was produced, where the *X*-axis was the time axis (the unit is the interval between frames), and the *Y*-axis was the distance along the ROI (the unit is the distance in pixels traveled by the tube apex). Speed was measured by the same plug-in. Video clips were recorded for at least 10 pollen tubes in the cases investigated.

### 5.9. Analysis of Generative Cell Movement

The test of generative cell movement was performed by supplying the dye 4′,6-diamidino-2-phenylindole (DAPI) to pollen tubes allowed to grow for at least 6 h. Pollen tubes were placed on slides and then stained with DAPI; after a few minutes of incubation, observations were made using a Zeiss AxioImager fluorescence microscope equipped with a 63× objective (Carl Zeiss AG, Jena, Germany). Images were captured by the MRm AxioCam camera as controlled by the AxioVision software. Total length of pollen tubes and distance covered by the generative cells from the pollen grain were measured with ImageJ.

### 5.10. Cloning of Expression Plasmids and Subcellular Localization of CcASP-RICH

Using Gateway technology (Invitrogen, Waltham, MA, USA) the gene of interest was cloned into the modified binary vector pB7WGR2,0 [55] using the cauliflower mosaic virus 35S promoter upstream of coding fusions to GFP or mCherry, respectively. *Nicotiana tabacum* (SR1 cv Petit Havana) plants 4 to 5 weeks old were used for transient protein expression of fluorescent constructs as described by [56]. In brief, transformed agrobacteria were pelleted by centrifugation, washed by infiltration buffer, and finally resuspended. The bacterial suspension was injected through the stomata on the underside of the tobacco leaf using a 1 mL syringe, and infiltrated plants were kept at 22 °C for 72 h prior to imaging.

### 5.11. Statistical Analysis

For *CcASP-RICH* expression; viability of pollen grains, germination rate, and tube length; distribution of actin filaments; and analysis of callose plug deposition; differences between sample sets were analyzed by analysis of variance (one-way ANOVA, with a threshold *p*-value of 0.05 using GraphPad Prism with the “anova” function, followed by a post hoc “pairwise.t.test” function, with thresholds of *p* = 0.05 and *p* = 0.01).

## Data Availability

Not applicable.

## Supplementary Materials

The following supporting information can be downloaded at: https://www.mdpi.com/article/10.3390/ijms23147880/s1.
