# Supplementary material for: Expression of Clementine Asp-Rich Proteins (CcASP-RICH) in Tobacco Plants Interferes with the Mechanism of Pollen Tube Growth
Source: Int J Mol Sci. 2022 Jul 17;23(14):7880. doi: 10.3390/ijms23147880 (PMC9316813; doi:10.3390/ijms23147880)
Supplement: Supplementary file 1 [file ijms-23-07880-s001.zip › ijms-1804468-supplementary.pdf]

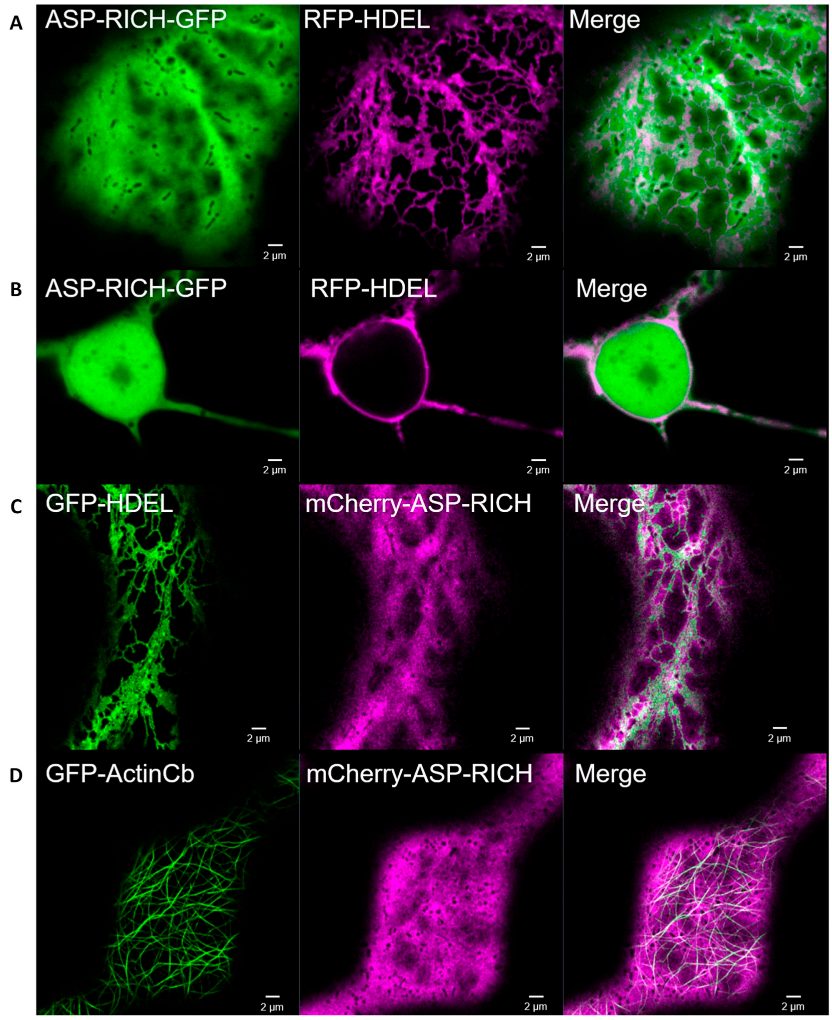

Figure S1: Subcellular localization of CcASP-RICH. CcASP-RICH-GFP or CcASP-RICH-mCherry were expressed transiently in N. tabacum and analyzed for localization and co-localization 3 days after infiltration. Fluorescence signals for CcASP-RICH-GFP or CcASP-RICH-mCherry were detected in the cytosol. Representative images are shown for CcASP-RICH-GFP coexpressed with the ER lumenal marker RFP-HDEL labelling the ER network (A) as well as the nuclear envelope (B). CcASP-RICH-mCherry was co-expressed with the ER marker GFP-HDEL (C) as well as the actin binding chromobody (ActinCb, D). Scale bar are given.

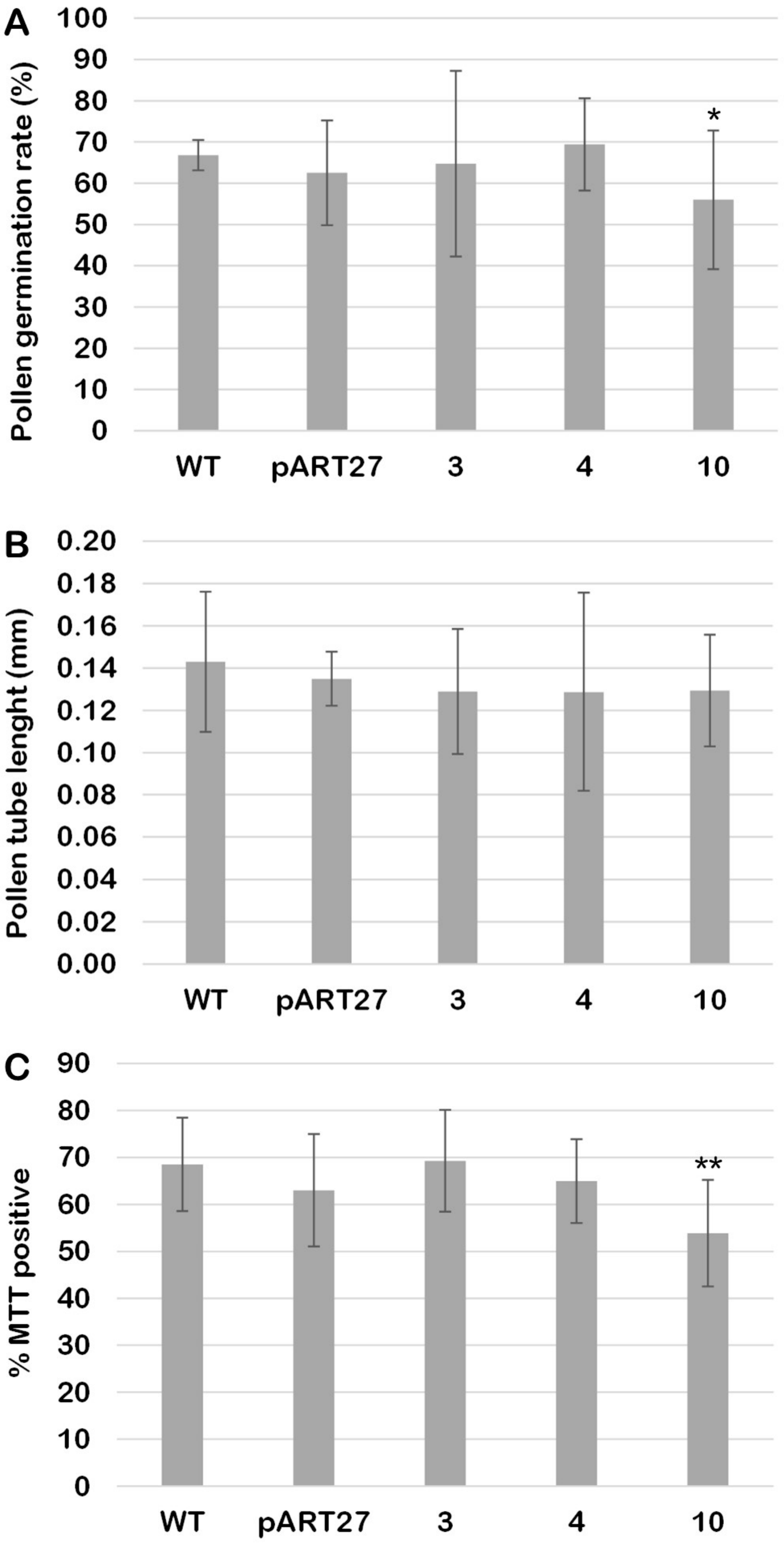

Figure S2: Viability of pollen grains, germination rate and tube length of at least 100 pollen grains for each line analyzed. (A) Germination rate, expressed as percentage, of pollen grains. (B), Length of pollen tubes after 3 hours of germination. (C), percentage of MTT positive pollen grains. Sample sets were compared with One-Way ANOVA. Asterisks indicate statistically significant differences (* = $p < 0.05$ and (** = $p < 0.01$).

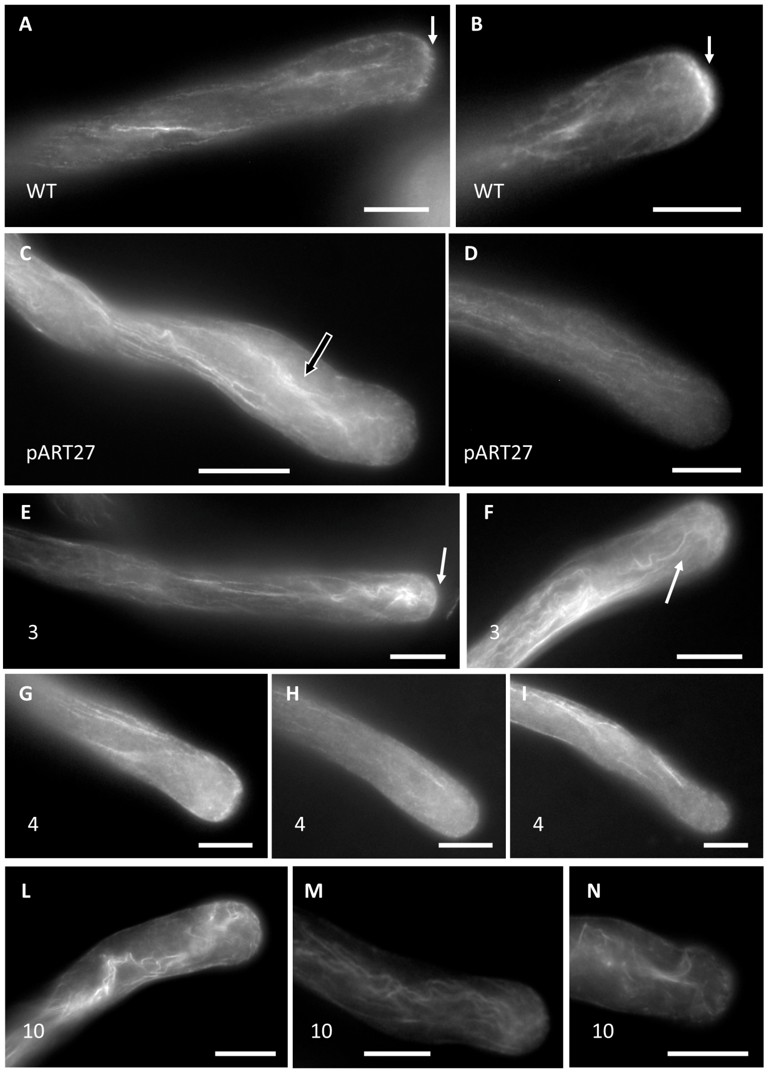

Figure S3: Distribution of actin filaments in WT pollen tubes and in pollen tubes expressing CcASP-RICH. (A, B) WT pollen tubes with the typical longitudinal distribution of actin filaments and actin fringe (arrows). (C, D) Pollen tubes expressing pART27 with a longitudinal array of actin filaments (arrow). (E, F) Actin filaments in line 3. Several wavy and curved actin bundles are observable. Actin fringe is very rare. (G, H, I) Actin filaments in line 4. The fibrillar appearance of actin is perceptible although in several cases it is very weak; sometimes intense actin bundles are observable. (L, M, N) Actin filaments in line 10. Here, curved wavy bundles are observable indicating substantial damage to actin filaments. Bars: 10 μm.

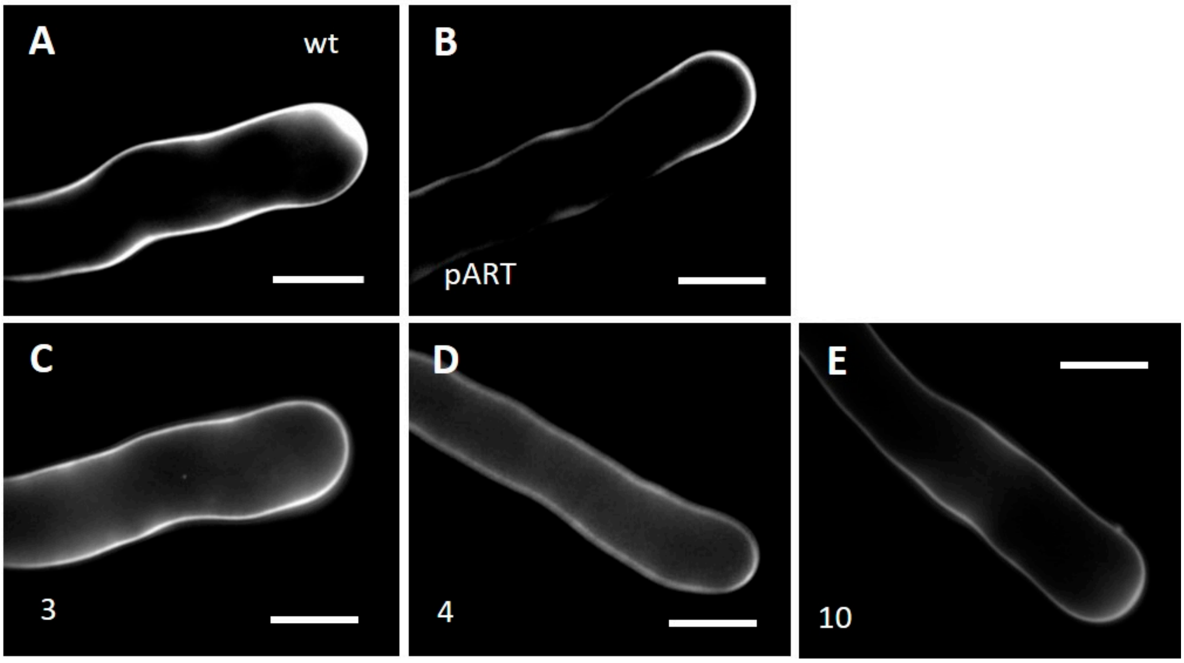

Figure S4: Distribution of newly secreted cell wall material. (A) WT pollen tube with accumulation of cell wall material at the apex. (B) Pollen tube expressing pART27 shows fluorescence signal at the apex. (C) Pollen tube of line 3 with more homogeneous distribution of new cell wall material, interspersed with fluorescence peaks. (D) Pollen tube of line 4 showing distribution of newly secreted cell wall material similar to line 3. (E) Pollen tube of line 10 showing homogeneous distribution of new cell wall material. Bars: 10 μm.

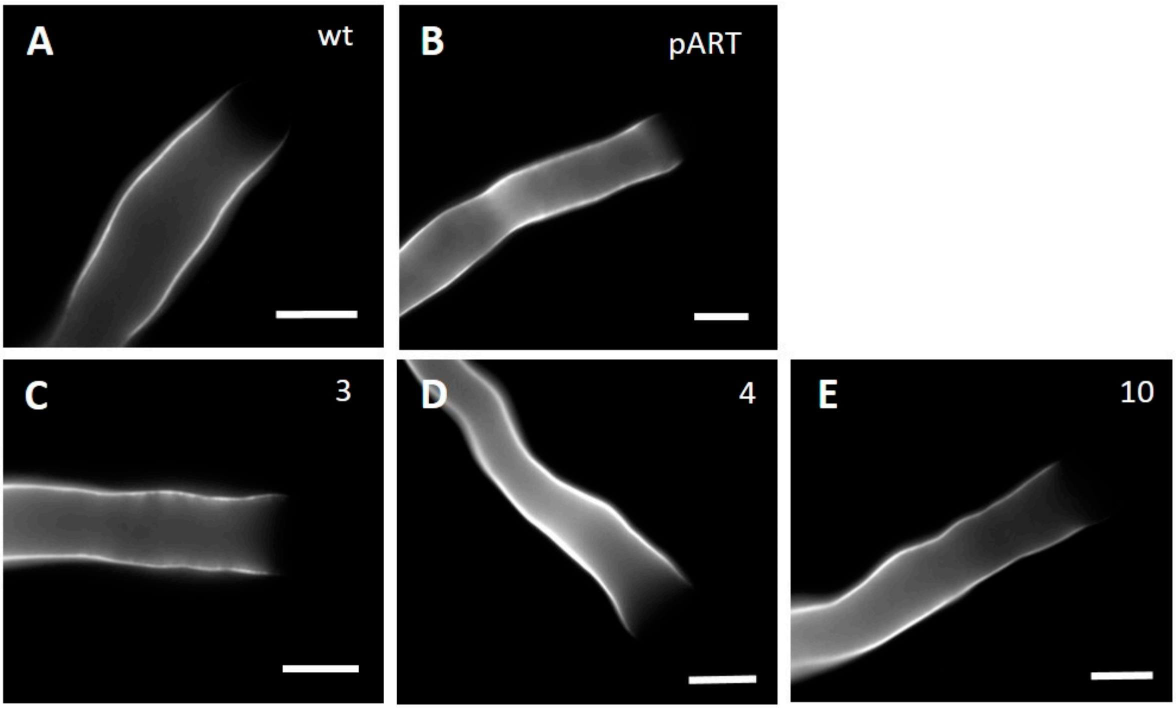

Figure S5: Distribution of callose in WT and transgenic pollen tubes. (A) A WT pollen tube with accumulation of callose in shank and absence in the apex. (B) A pollen tube expressing pART27. The pattern of callose is similar to the control. (C) Pollen tube of line 3. Callose is absent in the apex but is uniform in shank. (D) Pollen tube of line 4. Again, there is no callose in the apex but and evenly distribution in shank. (E) Pollen tube of line 10. Callose is absent in the apex and is evenly distributed in shank. Bars: 10 μm. In all cases, a very weak signal was found in the first 5-10 μm from the apex then the amount of callose increased progressively, although with different trends in distinct samples.
